# *m*-Xylylenediaminium di­aqua­bis­[di­hydrogen diphosphato(2−)]cobaltate(II) dihydrate

**DOI:** 10.1107/S160053681302535X

**Published:** 2013-09-18

**Authors:** Adel Elboulali, Samah Akriche, Mohamed Rzaigui

**Affiliations:** a Laboratoire de Chimie des Matériaux, Faculté des Sciences de Bizerte, 7021 Zarzouna Bizerte, Tunisie

## Abstract

In the title complex, (C_8_H_14_N_2_)[Co(H_2_P_2_O_7_)_2_(H_2_O)_2_]·2H_2_O, the Co^II^ ion lies on an inversion center and is coordinated by two bidentate diphosphate ligands and two water mol­ecules in a slightly distorted octa­hedral coordination geometry. The *m*-xylylenediaminium cation is located on a twofold rotation axis. In the crystal, a three-dimensional supra­molecular assembly is constructed by O—H⋯O and N—H⋯O hydrogen bonds between the organic cations, complex anions and uncoordin­ated water mol­ecules.

## Related literature

For applications of diphosphate compounds containing trans­ition metals, see: Erragh *et al.* (1998 ▶); Handizi *et al.* (1994 ▶); Dridi *et al.* (2000 ▶); Cheetham *et al.* (1999 ▶); Clearfield (1998 ▶). For bond-valence-sum calculations, see: Brown & Altermatt (1985 ▶). For geometrical features in related structures, see: Selmi *et al.* (2006*a* ▶,*b* ▶, 2009 ▶); Gharbi *et al.* (1994 ▶); Gharbi & Jouini (2004 ▶); Nelson *et al.* (2007 ▶).
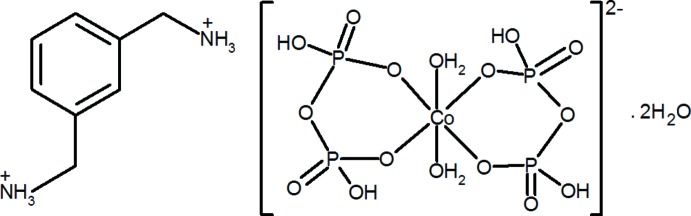


## Experimental

### 

#### Crystal data

(C_8_H_14_N_2_)[Co(H_2_P_2_O_7_)_2_(H_2_O)_2_]·2H_2_O
*M* *_r_* = 621.12
Monoclinic, 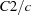

*a* = 11.933 (2) Å
*b* = 9.132 (4) Å
*c* = 21.441 (3) Å
β = 101.20 (2)°
*V* = 2291.8 (11) Å^3^
*Z* = 4
Ag *K*α radiation
λ = 0.56087 Å
μ = 0.58 mm^−1^
*T* = 293 K
0.27 × 0.21 × 0.15 mm

#### Data collection

Enraf Nonius CAD4 diffractometer
7386 measured reflections
5609 independent reflections
4197 reflections with *I* > 2σ(*I*)
*R*_int_ = 0.019
2 standard reflections every 120 min intensity decay: 2%

#### Refinement

*R*[*F* ^2^ > 2σ(*F* ^2^)] = 0.035
*wR*(*F* ^2^) = 0.090
*S* = 1.06
5609 reflections
167 parameters
6 restraints
H atoms treated by a mixture of independent and constrained refinement
Δρ_max_ = 0.87 e Å^−3^
Δρ_min_ = −0.41 e Å^−3^

### 

Data collection: *CAD-4 EXPRESS* (Enraf–Nonius, 1994 ▶); cell refinement: *CAD-4 EXPRESS*; data reduction: *XCAD4* (Harms & Wocadlo, 1996 ▶); program(s) used to solve structure: *SHELXS86* (Sheldrick, 2008 ▶); program(s) used to refine structure: *SHELXL97* (Sheldrick, 2008 ▶); molecular graphics: *ORTEP-3 for Windows* (Farrugia, 2012 ▶) and *DIAMOND* (Brandenburg & Putz, 2005 ▶); software used to prepare material for publication: *WinGX* publication routines (Farrugia, 2012 ▶).

## Supplementary Material

Crystal structure: contains datablock(s) I. DOI: 10.1107/S160053681302535X/lh5650sup1.cif

Structure factors: contains datablock(s) I. DOI: 10.1107/S160053681302535X/lh5650Isup2.hkl

Additional supplementary materials:  crystallographic information; 3D view; checkCIF report

## Figures and Tables

**Table 1:** Hydrogen-bond geometry (Å, °)

*D*—H⋯*A*	*D*—H	H⋯*A*	*D*⋯*A*	*D*—H⋯*A*
O2—H2*O*2⋯O6^i^	0.82	1.74	2.5574 (18)	172
O7—H7⋯O3^ii^	0.82	1.74	2.5268 (18)	160
O1*W*—H1*W*1⋯O6^i^	0.84 (1)	1.99 (1)	2.8289 (17)	174 (2)
O1*W*—H2*W*1⋯O3^iii^	0.85 (1)	1.94 (1)	2.7891 (17)	174 (2)
O2*W*—H1*W*2⋯O3^iv^	0.85 (1)	2.15 (1)	2.972 (2)	162 (2)
O2*W*—H2*W*2⋯O6^v^	0.86 (1)	2.12 (1)	2.946 (2)	163 (2)
N1—H1*A*⋯O2^i^	0.89	2.22	2.9694 (18)	142
N1—H1*A*⋯O2*W* ^vi^	0.89	2.36	2.969 (3)	126
N1—H1*B*⋯O7^iii^	0.89	2.01	2.8893 (18)	167
N1—H1*C*⋯O5	0.89	1.99	2.8701 (19)	171

Symmetry codes: (i) 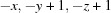; (ii) 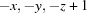; (iii) 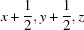; (iv) 
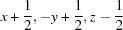
; (v) 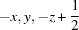; (vi) 
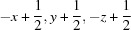
.

**Fig. 1.**
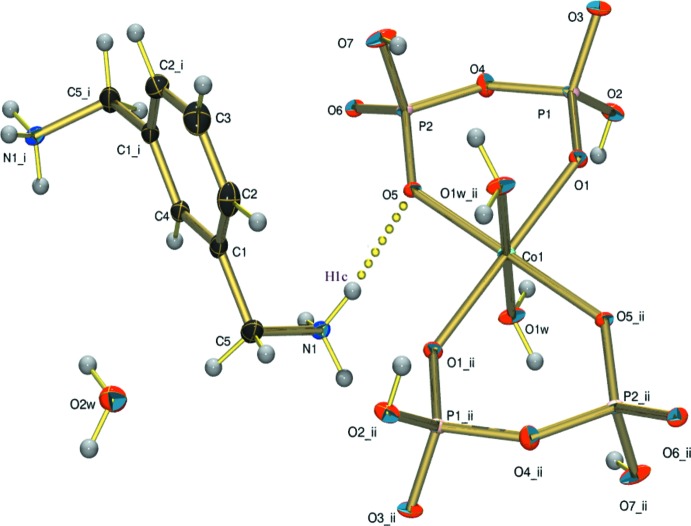
An ORTEP view of (I) with displacement ellipsoids drawn at the 30% probability level. H atoms are represented as small spheres of arbitrary radii. A hydrogen bond is represented as a dotted line [Symmetry codes: (i) -x, y, -z+1/2; (ii) -x+1/2, -y+1/2, -z+1].

**Fig. 2.**
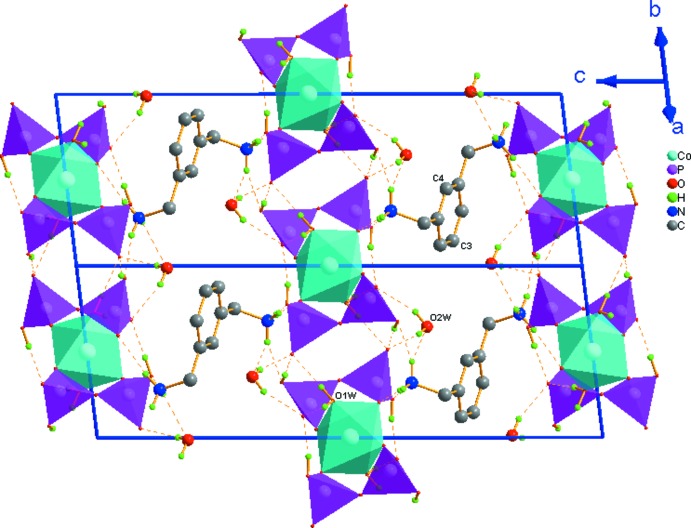
A projection of (I) along the [110] direction. The H-atoms not involved in H-bonding are omitted. Hydrogen bonds are shown as dashed lines.

## supplementary crystallographic information

### 1. Comment

Among a variety of organic inorganic hybrid materials, diphosphate compounds containing transition metals showed promising properties in diverse areas such as catalysis (Erragh *et al.*, 1998), magnetism (Handizi *et al.*, 1994), conductivity (Dridi *et al.*, 2000), ion-exchange or second-order non-linear optics (Cheetham *et al.*, 1999; Clearfield, 1998). Here, we report a new diphosphate of mixed organic-metal cations: (C_8_H_14_N_2_)[Co(H_2_P_2_O_7_)_2_(H_2_O)_2_]·2(H_2_O) (I). The asymmetric unit of (I) is made up of a half of mononuclear [Co(H_2_P_2_O_7_)_2_(H_2_O)_2_]^2-^ moiety, a half of organic cation and one water of crystallization. As the Co^II^ ion and C3 and C4 atoms are located respectively on inversion center and twofold rotation axis, the complete formula unit is generated by these crystallographic elements of symmetry (Fig. 1).

Each Co^II^ ion is coordinated by four oxygen atoms from two chelating diphosphate ligands and two oxygen atoms from two coordinated (O1W) water molecules to form a slightly distorted CoO_6_ octahedron. The valence bond calculation (Brown & Altermatt, 1985) based on these six oxygen distances gives an effective bond valence of 2.0185 consistent with the cationic charge of +2. The bond lengths and angles around the Co^II^ ion 2.0695 (11)—2.1044 (11) Å (Co—O) and 85.99 (5)—180.00 (8)° (O—Co—O) are close to those reported for Co metals in (C_9_H_11_NH_3_)_2_[Co(H_2_P_2_O_7_)_2_(H_2_O)_2_] (Selmi *et al.*, 2006*a*), (C_8_H_12_N)_2_[Co(H_2_P_2_O_7_)_2_(H_2_O)_2_] (Selmi *et al.*, 2006*b*) and (C_7_H_10_N)_2_[Co(H_2_P_2_O_7_)_2_(H_2_O)_2_] (Selmi *et al.*, 2009) in related structures. The discrete CoO_6_ entities are isolated in the structure with Co···Co separations of over 7 Å. In addition, the chelating P_2_O_7_ group has a quasi-eclipsed conformation with O—P—P—O torsion angles averaging 18.8 ° and bridges the Co atom through O1—P1 and O5—P2 linkages thus producing a bent P_2_O_7_ group, with a P1—O4—P2 angle of 132.91 (7)° as observed in other M^II^–organic diphosphate frameworks (Selmi *et al.*, 2006*a*, 2006*b* and 2009; Gharbi *et al.*, 2004,1994). With regards to the geometrical features of organic cations, the main bond lenghts are comparable to those observed in the *p*-xylylenediaminium cations in {[C_8_H_14_N_2_]_3_[Mo_9_O_30_]·2H_2_O}_n_ (Nelson *et al.*, 2007).

As shown in Fig.2 and reported in Table 1, the [Co(H_2_P_2_O_7_)_2_(H_2_O)_2_]^2-^ clusters are interconnected *via* O—H···O hydrogen bonding interactions involving the hydroxyl groups of [H_2_P_2_O_7_]^2-^ and OW1 water molecules into anionic layers along *c*-axis at *z* = 0 and 1/2. The remaining uncoordinated O2W water molecules further link these layers so as to contribute to their cohesion with O···O separations ranging from 2.946 (2)to 2.972 (2) Å (Table 1). The so-obtained two-dimensional-subnetworks stack together by means NH_3_ groups of the diprotonated *m*-xylylenediaminium cations via moderate N—H···O hydrogen bonds (mean N···O = 2.924 Å, Table 1) and electrostatic interactions so as to build a three-dimensional supramolecular network.

### 2. Experimental

Pink prismatic shaped crystals of the title compound were synthesized by the reaction of diphosphoric acid H_4_P_2_O_7_ (2 mmol), CoCl_2_·6H_2_O (0.24 g; 1 mmol)and *m*-xylylenediamine (0.14 g; 1 mmol) carried out in water–ethanol (5:1) at rt. The diphosphoric acid, H_4_P_2_O_7_, was obtained from Na_4_P_2_O_7_ by using an ion-exchange resin (Amberlite IR 120).

### 3. Refinement

All H atoms attached to C, O and N atoms were fixed geometrically and treated as riding, with C—H = 0.93 Å with *U*_iso_(H) = 1.2U_eq_(C) for the aromatic ring and C—H = 0.97 Å and N—H = 0.89 Å respectively for CH_2_ and NH_3_ cation groups and O—H = 0.82 Å for diphosphoric anion with *U*_iso_(H) = 1.5U_eq_(C, O or N). The water H atoms were refined using restraints [O—H = 0.85 (1) A °, H···H = 1.44 (2) A ° and *U*_iso_(H) = 1.5U_eq_(O)].

### Crystal data

**Table:**

(C_8_H_14_N_2_)[Co(H_2_P_2_O_7_)_2_(H_2_O)_2_]·2H_2_O	*F*(000) = 1276
*M**_r_* = 621.12	*D*_x_ = 1.800 Mg m^−^^3^
Monoclinic, *C*2/*c*	Ag *K*α radiation, λ = 0.56087 Å
Hall symbol: -C 2yc	Cell parameters from 25 reflections
*a* = 11.933 (2) Å	θ = 9–11°
*b* = 9.132 (4) Å	µ = 0.58 mm^−^^1^
*c* = 21.441 (3) Å	*T* = 293 K
β = 101.20 (2)°	Prism, pink
*V* = 2291.8 (11) Å^3^	0.27 × 0.21 × 0.15 mm
*Z* = 4	

### Data collection

**Table:**

Enraf Nonius CAD4 diffractometer	*R*_int_ = 0.019
Radiation source: fine-focus sealed tube	θ_max_ = 28.0°, θ_min_ = 2.2°
Graphite monochromator	*h* = −19→19
non–profiled ω scans	*k* = −2→15
7386 measured reflections	*l* = −2→35
5609 independent reflections	2 standard reflections every 120 min
4197 reflections with *I* > 2σ(*I*)	intensity decay: 2%

### Refinement

**Table:**

Refinement on *F*^2^	Primary atom site location: structure-invariant direct methods
Least-squares matrix: full	Secondary atom site location: difference Fourier map
*R*[*F*^2^ > 2σ(*F*^2^)] = 0.035	Hydrogen site location: inferred from neighbouring sites
*wR*(*F*^2^) = 0.090	H atoms treated by a mixture of independent and constrained refinement
*S* = 1.06	*w* = 1/[σ^2^(*F*_o_^2^) + (0.0412*P*)^2^ + 1.174*P*] where *P* = (*F*_o_^2^ + 2*F*_c_^2^)/3
5609 reflections	(Δ/σ)_max_ = 0.001
167 parameters	Δρ_max_ = 0.87 e Å^−^^3^
6 restraints	Δρ_min_ = −0.41 e Å^−^^3^

### Special details

**Table:**

Geometry. All e.s.d.'s (except the e.s.d. in the dihedral angle between two l.s. planes) are estimated using the full covariance matrix. The cell e.s.d.'s are taken into account individually in the estimation of e.s.d.'s in distances, angles and torsion angles; correlations between e.s.d.'s in cell parameters are only used when they are defined by crystal symmetry. An approximate (isotropic) treatment of cell e.s.d.'s is used for estimating e.s.d.'s involving l.s. planes.
Refinement. Refinement of *F*^2^ against ALL reflections. The weighted *R*-factor *wR* and goodness of fit *S* are based on *F*^2^, conventional *R*-factors *R* are based on *F*, with *F* set to zero for negative *F*^2^. The threshold expression of *F*^2^ > σ(*F*^2^) is used only for calculating *R*-factors(gt) *etc*. and is not relevant to the choice of reflections for refinement. *R*-factors based on *F*^2^ are statistically about twice as large as those based on *F*, and *R*- factors based on ALL data will be even larger.

### Fractional atomic coordinates and isotropic or equivalent isotropic displacement parameters (Å^2^)

**Table:**

	*x*	*y*	*z*	*U*_iso_*/*U*_eq_	
Co1	0.2500	0.2500	0.5000	0.01705 (6)	
P1	0.02843 (3)	0.22128 (4)	0.571350 (18)	0.01809 (7)	
P2	−0.02421 (3)	0.29251 (4)	0.435477 (17)	0.01764 (7)	
O1	0.14860 (8)	0.19560 (13)	0.56463 (5)	0.0244 (2)	
O2	0.01855 (10)	0.34054 (12)	0.62180 (6)	0.0295 (2)	
H2O2	0.0510	0.4153	0.6137	0.044*	
O3	−0.03555 (9)	0.08962 (12)	0.58755 (6)	0.0272 (2)	
O4	−0.04173 (9)	0.29279 (16)	0.50765 (6)	0.0343 (3)	
O5	0.10009 (8)	0.30371 (12)	0.43469 (5)	0.02264 (19)	
O6	−0.10026 (8)	0.41352 (11)	0.40494 (5)	0.0240 (2)	
O7	−0.07639 (9)	0.14643 (13)	0.40722 (7)	0.0373 (3)	
H7	−0.0275	0.0822	0.4135	0.056*	
O1W	0.25749 (9)	0.47229 (13)	0.52445 (7)	0.0317 (3)	
H1W1	0.2077 (14)	0.501 (3)	0.5446 (10)	0.050*	
H2W1	0.3230 (10)	0.503 (3)	0.5427 (10)	0.050*	
O2W	0.31653 (13)	0.3286 (2)	0.17868 (8)	0.0503 (4)	
H1W2	0.3666 (14)	0.365 (3)	0.1596 (10)	0.050*	
H2W2	0.2488 (10)	0.355 (3)	0.1618 (10)	0.050*	
N1	0.20006 (11)	0.51928 (16)	0.36481 (7)	0.0286 (3)	
H1A	0.1506	0.5933	0.3594	0.043*	
H1B	0.2686	0.5518	0.3836	0.043*	
H1C	0.1768	0.4511	0.3891	0.043*	
C1	0.09999 (15)	0.3728 (2)	0.27477 (8)	0.0336 (3)	
C2	0.0986 (2)	0.2211 (3)	0.27536 (10)	0.0499 (5)	
H2	0.1644	0.1696	0.2929	0.060*	
C3	0.0000	0.1464 (4)	0.2500	0.0578 (9)	
H3	0.0000	0.0446	0.2500	0.069*	
C4	0.0000	0.4479 (3)	0.2500	0.0305 (4)	
H4	0.0000	0.5497	0.2500	0.037*	
C5	0.20697 (16)	0.4554 (3)	0.30225 (10)	0.0477 (5)	
H5A	0.2719	0.3896	0.3070	0.057*	
H5B	0.2186	0.5330	0.2733	0.057*	

### Atomic displacement parameters (Å^2^)

**Table:**

	*U*^11^	*U*^22^	*U*^33^	*U*^12^	*U*^13^	*U*^23^
Co1	0.01044 (9)	0.01735 (11)	0.02374 (12)	0.00015 (8)	0.00423 (8)	−0.00035 (9)
P1	0.01487 (12)	0.01621 (14)	0.02461 (16)	0.00092 (10)	0.00736 (11)	0.00057 (11)
P2	0.01156 (12)	0.01553 (14)	0.02536 (16)	0.00003 (10)	0.00241 (11)	0.00060 (12)
O1	0.0148 (4)	0.0313 (5)	0.0283 (5)	0.0045 (4)	0.0073 (4)	0.0064 (4)
O2	0.0363 (6)	0.0195 (5)	0.0374 (6)	−0.0048 (4)	0.0187 (5)	−0.0060 (4)
O3	0.0217 (4)	0.0178 (4)	0.0446 (6)	−0.0032 (4)	0.0129 (4)	−0.0008 (4)
O4	0.0202 (4)	0.0539 (8)	0.0304 (6)	0.0138 (5)	0.0085 (4)	0.0100 (5)
O5	0.0124 (3)	0.0297 (5)	0.0256 (5)	−0.0013 (3)	0.0032 (3)	0.0023 (4)
O6	0.0182 (4)	0.0184 (4)	0.0339 (5)	0.0032 (3)	0.0014 (4)	0.0034 (4)
O7	0.0193 (5)	0.0182 (5)	0.0697 (9)	−0.0006 (4)	−0.0025 (5)	−0.0103 (5)
O1W	0.0178 (4)	0.0256 (5)	0.0525 (7)	−0.0017 (4)	0.0090 (5)	−0.0130 (5)
O2W	0.0372 (7)	0.0640 (11)	0.0506 (9)	−0.0005 (7)	0.0106 (7)	0.0063 (8)
N1	0.0203 (5)	0.0316 (7)	0.0327 (7)	−0.0009 (5)	0.0021 (5)	0.0041 (5)
C1	0.0308 (7)	0.0460 (10)	0.0233 (7)	0.0031 (7)	0.0037 (6)	−0.0020 (7)
C2	0.0589 (13)	0.0493 (12)	0.0396 (10)	0.0199 (10)	0.0051 (9)	0.0030 (9)
C3	0.086 (3)	0.0330 (14)	0.0521 (19)	0.000	0.0083 (18)	0.000
C4	0.0304 (10)	0.0333 (12)	0.0271 (10)	0.000	0.0041 (8)	0.000
C5	0.0262 (8)	0.0832 (17)	0.0347 (9)	−0.0055 (9)	0.0088 (7)	−0.0058 (10)

### Geometric parameters (Å, º)

**Table:**

Co1—O1^i^	2.0695 (11)	O2W—H1W2	0.853 (9)
Co1—O1	2.0695 (11)	O2W—H2W2	0.855 (9)
Co1—O1W^i^	2.0940 (15)	N1—C5	1.480 (3)
Co1—O1W	2.0940 (14)	N1—H1A	0.8900
Co1—O5	2.1044 (11)	N1—H1B	0.8900
Co1—O5^i^	2.1044 (11)	N1—H1C	0.8900
P1—O1	1.4873 (10)	C1—C2	1.385 (3)
P1—O3	1.5007 (12)	C1—C4	1.389 (2)
P1—O2	1.5554 (12)	C1—C5	1.501 (3)
P1—O4	1.5965 (12)	C2—C3	1.377 (3)
P2—O5	1.4901 (10)	C2—H2	0.9300
P2—O6	1.4975 (11)	C3—C2^ii^	1.377 (3)
P2—O7	1.5452 (13)	C3—H3	0.9300
P2—O4	1.6012 (13)	C4—C1^ii^	1.389 (2)
O2—H2O2	0.8200	C4—H4	0.9300
O7—H7	0.8200	C5—H5A	0.9700
O1W—H1W1	0.843 (9)	C5—H5B	0.9700
O1W—H2W1	0.849 (9)		
			
O1^i^—Co1—O1	180.0	P2—O5—Co1	134.09 (7)
O1^i^—Co1—O1W^i^	93.85 (5)	P2—O7—H7	109.5
O1—Co1—O1W^i^	86.15 (5)	Co1—O1W—H1W1	115.7 (17)
O1^i^—Co1—O1W	86.15 (5)	Co1—O1W—H2W1	114.9 (17)
O1—Co1—O1W	93.85 (5)	H1W1—O1W—H2W1	109.9 (18)
O1W^i^—Co1—O1W	180.00 (8)	H1W2—O2W—H2W2	112.3 (18)
O1^i^—Co1—O5	91.75 (4)	C5—N1—H1A	109.5
O1—Co1—O5	88.25 (4)	C5—N1—H1B	109.5
O1W^i^—Co1—O5	94.01 (5)	H1A—N1—H1B	109.5
O1W—Co1—O5	85.99 (5)	C5—N1—H1C	109.5
O1^i^—Co1—O5^i^	88.25 (4)	H1A—N1—H1C	109.5
O1—Co1—O5^i^	91.75 (4)	H1B—N1—H1C	109.5
O1W^i^—Co1—O5^i^	85.99 (5)	C2—C1—C4	119.07 (19)
O1W—Co1—O5^i^	94.01 (5)	C2—C1—C5	120.64 (19)
O5—Co1—O5^i^	180.00 (5)	C4—C1—C5	120.3 (2)
O1—P1—O3	116.07 (7)	C3—C2—C1	120.2 (2)
O1—P1—O2	112.49 (7)	C3—C2—H2	119.9
O3—P1—O2	106.82 (7)	C1—C2—H2	119.9
O1—P1—O4	109.69 (6)	C2^ii^—C3—C2	120.6 (3)
O3—P1—O4	108.66 (7)	C2^ii^—C3—H3	119.7
O2—P1—O4	102.15 (7)	C2—C3—H3	119.7
O5—P2—O6	117.67 (6)	C1^ii^—C4—C1	120.8 (2)
O5—P2—O7	112.39 (7)	C1^ii^—C4—H4	119.6
O6—P2—O7	107.56 (7)	C1—C4—H4	119.6
O5—P2—O4	109.15 (7)	N1—C5—C1	111.18 (15)
O6—P2—O4	103.86 (7)	N1—C5—H5A	109.4
O7—P2—O4	105.19 (8)	C1—C5—H5A	109.4
P1—O1—Co1	136.65 (7)	N1—C5—H5B	109.4
P1—O2—H2O2	109.5	C1—C5—H5B	109.4
P1—O4—P2	132.91 (7)	H5A—C5—H5B	108.0
			
O3—P1—O1—Co1	132.63 (10)	O7—P2—O5—Co1	−91.76 (11)
O2—P1—O1—Co1	−103.92 (11)	O4—P2—O5—Co1	24.54 (12)
O4—P1—O1—Co1	9.04 (13)	O1^i^—Co1—O5—P2	175.43 (10)
O1^i^—Co1—O1—P1	112.8 (17)	O1—Co1—O5—P2	−4.57 (10)
O1W^i^—Co1—O1—P1	−109.69 (11)	O1W^i^—Co1—O5—P2	81.45 (10)
O1W—Co1—O1—P1	70.31 (11)	O1W—Co1—O5—P2	−98.55 (10)
O5—Co1—O1—P1	−15.56 (11)	O5^i^—Co1—O5—P2	−39 (100)
O5^i^—Co1—O1—P1	164.44 (11)	C4—C1—C2—C3	1.9 (3)
O1—P1—O4—P2	23.46 (15)	C5—C1—C2—C3	−179.98 (16)
O3—P1—O4—P2	−104.38 (13)	C1—C2—C3—C2^ii^	−0.95 (14)
O2—P1—O4—P2	142.97 (12)	C2—C1—C4—C1^ii^	−0.93 (14)
O5—P2—O4—P1	−38.26 (15)	C5—C1—C4—C1^ii^	−179.09 (18)
O6—P2—O4—P1	−164.55 (12)	C2—C1—C5—N1	−104.4 (2)
O7—P2—O4—P1	82.55 (13)	C4—C1—C5—N1	73.8 (2)
O6—P2—O5—Co1	142.47 (8)		

Symmetry codes: (i) −*x*+1/2, −*y*+1/2, −*z*+1; (ii) −*x*, *y*, −*z*+1/2.

### Hydrogen-bond geometry (Å, º)

**Table:**

*D*—H···*A*	*D*—H	H···*A*	*D*···*A*	*D*—H···*A*
O2—H2*O*2···O6^iii^	0.82	1.74	2.5574 (18)	172
O7—H7···O3^iv^	0.82	1.74	2.5268 (18)	160
O1*W*—H1*W*1···O6^iii^	0.84 (1)	1.99 (1)	2.8289 (17)	174 (2)
O1*W*—H2*W*1···O3^v^	0.85 (1)	1.94 (1)	2.7891 (17)	174 (2)
O2*W*—H1*W*2···O3^vi^	0.85 (1)	2.15 (1)	2.972 (2)	162 (2)
O2*W*—H2*W*2···O6^ii^	0.86 (1)	2.12 (1)	2.946 (2)	163 (2)
N1—H1*A*···O2^iii^	0.89	2.22	2.9694 (18)	142
N1—H1*A*···O2*W*^vii^	0.89	2.36	2.969 (3)	126
N1—H1*B*···O7^v^	0.89	2.01	2.8893 (18)	167
N1—H1*C*···O5	0.89	1.99	2.8701 (19)	171

Symmetry codes: (ii) −*x*, *y*, −*z*+1/2; (iii) −*x*, −*y*+1, −*z*+1; (iv) −*x*, −*y*, −*z*+1; (v) *x*+1/2, *y*+1/2, *z*; (vi) *x*+1/2, −*y*+1/2, *z*−1/2; (vii) −*x*+1/2, *y*+1/2, −*z*+1/2.
